# Melatonin in Tuberous Sclerosis Complex Analysis Using Modern Mathematical Modeling Methods

**DOI:** 10.1155/2017/8234502

**Published:** 2017-04-25

**Authors:** Justyna Paprocka, Marek Kijonka, Łukasz Boguszewicz, Maria Sokół

**Affiliations:** ^1^Department of Pediatric Neurology, School of Medicine in Katowice, Medical University of Silesia, Katowice, Poland; ^2^Department of Medical Physics, Maria Skłodowska-Curie Memorial Cancer Center and Institute of Oncology, Gliwice Branch, Gliwice, Poland

## Abstract

*Purpose*. The aim of the study was to assess melatonin secretion pattern in children with TSC and to compare it with the secretion patterns in children with and without epilepsy. *Material and Methods*. Melatonin secretion was measured every three hours using the RIA method in four children with recognized TSC. The parameters of the melatonin secretion models were interpreted and compared with those obtained for the patients with epilepsy (*n* = 76) and the children from the control, nonepileptic group (*n* = 36). To describe the diurnal melatonin secretion, mathematical model was constructed and nonlinear least squares method with the Levenberg-Marquardt optimization algorithm was applied to approximate its parameters. The dim light melatonin onset (DLMO) parameters were also estimated from the model. *Results and Conclusions*. Statistically significant differences were found between the TSC melatonin secretion profiles and the nonepileptic control group. The profiles for the epileptic and TSC groups were found to be similar. For the TSC group, though a small one, the variations in the MLT release amplitudes seem to be independent of the total number of seizures; however, the MLT release shift appears to depend on the number of seizures.

## 1. Introduction

Tuberous sclerosis complex (TSC) affects 1 in 6000 live births. It is caused by mutation in a tumor suppressor gene: either the *TSC1* gene on chromosome 9 or the *TSC2* gene on chromosome 16; however, larger “genomic” mutations are very rare in TSC1 and more common in TSC2, occurring in about 6% of all TSC patients [1]. A mutation can be found in 85% of patients, and the total number of unique DNA variants of over 850 and 2400 for TSC1 and TSC2, respectively, has been shown to cause TSC up to date (Tuberous Sclerosis Database, http://chromium.liacs.nl/LOVD2/TSC/home.php). About 85%–90% of children and adolescents with TSC have CNS symptoms including epilepsy, cognitive impairment, behavioral problems, and autism-like symptoms [1, 2]. Epilepsy usually begins during the first year of life, frequently with focal seizures, tonic clonic or myoclonic seizures, or epileptic spasms. TSC patients may experience all kinds of seizures, which can become intractable over time [1]. Mutations in the *TSC1* or *TSC2* genes lead to disruption of the TSC1–TSC2 intracellular protein complex. At the cellular level, loss of TSC1 or TSC2 results in upregulation of the mammalian target of rapamycin (mTOR) protein complex [3]. The recognition of the role of mTOR pathway upregulation in TSC-associated lesions opens new possibilities for treatment strategy. Probably, mTOR inhibitors may not only suppress seizures, but also influence/reduce the epileptogenesis [4].

The etiology of sleep problems, frequently observed in TSC, remains unclear [5]. In children with TSC, severe sleep problems usually appear after the onset of epileptic spasms and are often due to sleep-related epileptic events (night waking, early waking, seizure-related sleep problems, and excessive daytime sleepiness) [5, 6]. Blunted melatonin (MLT) blood levels are especially interesting for their potential, yet unproven link with the disrupted sleep-wake cycle is frequently seen in many TSC children. In healthy subjects, melatonin secretion by the pineal gland increases prior to sleep onset and a peak is seen 4-5 hours after sleep onset. The circadian melatonin secretion in patients with epilepsy is characterized by an increased phase shift of melatonin release as compared to the nonepileptic patients [7]. Thus, since seizures are the most common neurological symptom of TSC, occurring in 96% children, similar disturbances may also be expected in TSC. However, to our knowledge, no one has looked at the circadian rhythms of melatonin production in children with TSC. In order to fill this literature gap, we decided to record the circadian melatonin secretion rhythms in TSC children and to analyze the data using the mathematical modelling proposed previously in our study of circadian rhythms of endogenous melatonin secretion in patients with epilepsy [7]. The parameters describing the diurnal melatonin secretion, melatonin concentration, release amplitude, phase shift of melatonin release, and sleep duration in children with TSC were compared with the data obtained for children with and without epilepsy. In addition, the mathematical model determines the dim light melatonin onset (DLMO) of melatonin secretion, as an important circadian marker [8].

## 2. Material and Methods

The study was approved by Silesian Medical University Ethics Committee. The informed written consents were taken from the parents or caregivers. The study was carried out at the Department of Pediatrics and Developmental Age Neurology of Medical University of Silesia in Katowice. None of the studied subjects had taken any medications affecting melatonin secretion (e.g., benzodiazepines and their agonists, fluvoxamine, caffeine, vitamin B12, nonsteroidal anti-inflammatory drugs: aspirin, ibuprofen, indometacin, adrenolytics, prostaglandins inhibitors, calcium channel blockers, dexamethason, clonidine, and antidepressants) before and during the study.

### 2.1. MLT Secretion Model

The methodology for determining individual parameters of melatonin cycle was based on our mathematical modeling of the diurnal melatonin secretion in children with epilepsy where the time dependencies of melatonin concentrations for each patient were approximated by the MLT(*t*) function [7]:

(1) $$\begin{array}{c} MLT\left(t\right)=b_{1}+b_{2}\exp\left(-\frac{{\left(\cos\left(\left(\pi /12\right)t-\left(\pi /12\right)b_{3}\right)-1\right)}^{2}}{{\left(\cos\left(\left(\pi /24\right)b_{4}\right)-1/\sqrt{\ln2}\right)}^{2}}\right). \end{array}$$

It is a modified Gaussian function [9] adopted to model the shape of the secretion cycle. Its period equals 24 hours, and the biophysical and clinical meaning of its four parameters *b*_1_–*b*_4_ are as follows [7]:

 
*b*_1_—minimum melatonin concentration (pg/mL),
 
*b*_2_—melatonin release amplitude (pg/mL),
 
*b*_3_—phase shift of melatonin release (h),
 
*b*_4_—sleep duration (represented by the full width at half maximum (FWHM) of melatonin secretion model) (h).

Maximum melatonin concentration, *b*_max_ (pg/mL), is given by a sum of *b*_1_ and *b*_2_.

The parameters describing the diurnal melatonin secretion were estimated using the Levenberg-Marquardt algorithm and the nonlinear least squares method.

The obtained parameters were also used to estimate the dim light melatonin onset. Due to the changes in the secretion patterns in pediatric patients, the relative thresholds (RT) were calculated from the melatonin profiles—as relative thresholds normalize differences in amplitude—thereby facilitating comparisons between differentiated individuals [10]. The threshold values for DLMO estimation were defined as the 25th (RT_25_) and 50th (RT_50_) percentiles of the maximum melatonin levels:

(2) $$\begin{array}{c} {RT}_{50}=0.5\cdot b_{\max},\\ {RT}_{25}=0.25\cdot b_{\max}. \end{array}$$

Then, the dim light melatonin onset and offset were calculated from the inverse function of (1) taking MLT(*t*) as an appropriate RT value. Consequently, for the 50% threshold, the DLMO onset equals

(3) $$\begin{array}{c} {DLMOon}_{50}=b_{3}-\frac{b_{4}}{2}, \end{array}$$

whereas the DLMO offset at the same threshold level (50%) is

(4) $$\begin{array}{c} {DLMOoff}_{50}=b_{3}+\frac{b_{4}}{2}. \end{array}$$

In case of the DLMO values at the 25% threshold level, the trigonometric parts do not reduce to simple expressions as for the 50% threshold ((3) and (4)). DLMOon_25_ is given by

(5) $$\begin{array}{c} {DLMOon}_{25}=b_{3}-\frac{12\cdot \arccos\left(1-\sqrt{2}+\sqrt{2}\cdot \cos\left(\left(\pi /24\right)b_{4}\right)\right)}{\pi }, \end{array}$$

whereas the DLMO offset of melatonin synthesis at the 25% threshold level is defined as

(6) $$\begin{array}{c} {DLMOoff}_{25}=b_{3}+\frac{12\cdot \arccos\left(1-\sqrt{2}+\sqrt{2}\cdot \cos\left(\left(\pi /24\right)b_{4}\right)\right)}{\pi }. \end{array}$$

Expressions (5) and (6) are quite complex; however, their trigonometric part

(7) $$\begin{array}{c} \frac{12\cdot \arccos\left(1-\sqrt{2}+\sqrt{2}\cdot \cos\left(\left(\pi /24\right)b_{4}\right)\right)}{\pi } \end{array}$$

can be approximated by a linear expression in the range of the argument *b*_4_ ∈ (0, 12), thus simplifying to

(8) $$\begin{array}{c} \frac{2}{\pi }b_{4}. \end{array}$$

Then, the approximated expressions for DLMOon_25_ and DLMOoff_25_ take the following forms:

(9) $$\begin{array}{c} {DLMOon}_{25}\approx b_{3}-\frac{2}{\pi }b_{4},\\ {DLMOoff}_{25}\approx b_{3}+\frac{2}{\pi }b_{4}. \end{array}$$

The calculated parameters *b*_1_ (minimum melatonin concentration), *b*_2_ (melatonin release amplitude), *b*_3_ (phase shift of melatonin release), *b*_4_ (full width at half maximum (FWHM) of melatonin secretion model), *b*_max_ (maximum melatonin concentration), DLMOon_50_ (DLMO onset at the 50% relative threshold), DLMOoff_50_ (DLMO offset at the 50% relative threshold), DLMOon_25_ (DLMO onset at the 25% relative threshold), and DLMOoff_25_ (DLMO offset at the 25% relative threshold) were subjected to statistical analysis, and the results were interpreted in view of the characteristics of melatonin secretion.

### 2.2. The Study Group

The study group (TSC-G) consists of 4 children with tuberous sclerosis complex at the age between 2 years 10 months and 7 years; mean age: 5 yrs 1.5 mo. The clinical characteristics of the TSC patients are available in Table 1. The patients were diagnosed based on the diagnostic TSC criteria revised at the International TSC Consensus Conference in 2012. All patients were treated from epilepsy (vigabatrin, valproic acid, and levetiracetam), and all suffered from delayed sleep onset or fragmented sleep, according to the parents' diaries.

### 2.3. The Epilepsy Group

The epilepsy group (E-G) included 76 patients (mean age: 6 years 9 months); female to male ratio was 40 : 36. The patients were reviewed for the seizure frequency, age at seizure onset, electroencephalogram tracings, current and previous AEDs, seizure timing, etiology, cognitive status, and family history. The type of epileptic seizures was defined following the International League Against Epilepsy Classification and Terminology. The mean duration of epilepsy was about 4.7 years (range: 2 months–17 years).

### 2.4. The Control Group

The control group (C-G) was constituted of 36 nonepileptic patients (mean age was 6 years and 11 months); female to male ratio was 21 : 15. Among the patients, peripheral nerve palsies (facial nerve palsy, *n* = 12, 33.3%; peroneal nerve palsy, *n* = 4, 11.1%), myopathy (*n* = 10, 27.7%), and back pain (*n* = 10, 27.7%) were diagnosed.

The E-G and C-G groups were also used in our previous publication [7]; however, in the current work, these two groups were increased by new cases.

### 2.5. Blood Sampling

Since the children with TSC were severely retarded, we decided to use blood as a material for the analyses.

The blood samples were drawn every 3 hours through an intravenous catheter. During night hours, blood samples were taken by red dim light. The melatonin level was analyzed using radioimmunoassay (RIA) method.

### 2.6. Statistical Tests

The statistical analysis was performed on the obtained melatonin model parameters. Since the individual group sizes differ markedly, and also the TSC-G group does not meet the requirements for a parametric test (the data is not normally distributed), in order to compare the TSC-G parameters with those obtained for the E-G and C-G groups, a nonparametric Mann-Whitney-Wilcoxon test was used.

The *p* values less than 0.05—a predetermined significance level—were accepted as indicating that the observed result would be highly unlikely under the null hypothesis. To explore the intragroup variability of TSC-G group, the qualitative research was also applied.

## 3. Results

**Figure 1:**
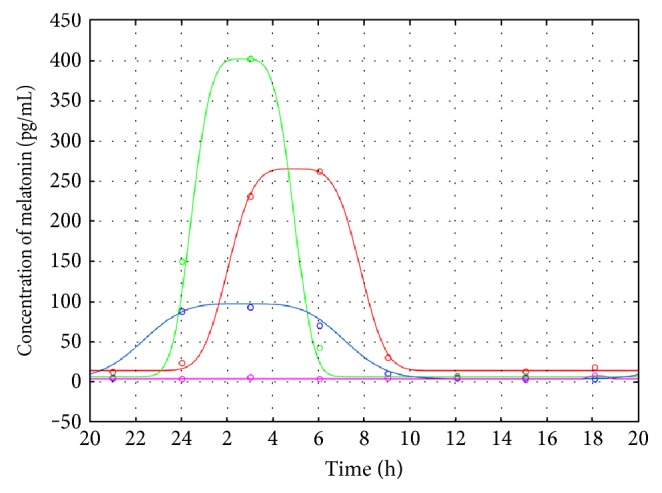
The melatonin profiles for the TSC patients: patient 1 (red line), patient 2 (green line), patient 3 (blue line), and patient 4 (magenta line). Measurement data are shown as colored circles.

The melatonin secretion curves were approximated for each patient separately, and the melatonin profiles for the TSC patients are shown in Figure 1.

**Figure 2:**
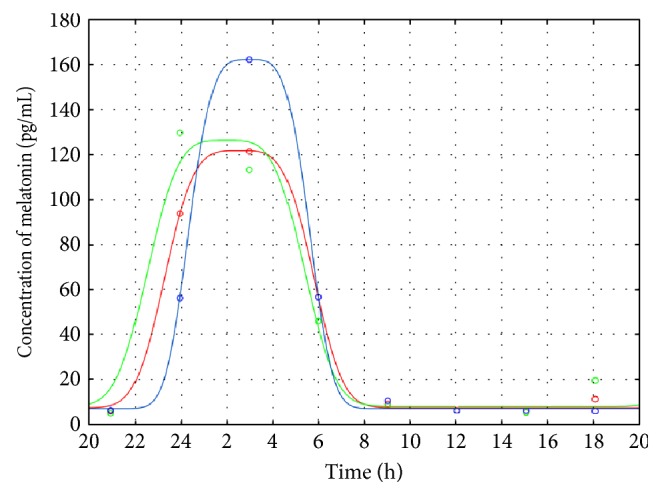
The average melatonin profiles for TSC-G (blue line), E-G (red line), and C-G (green line) obtained from the medians of the parameters. Median data points of specific time intervals are shown as colored circles.

In order to compare the secretion model parameters, the median profiles were also computed for TSC-G and for the epileptic (E-G) and nonepileptic (C-G) groups (Figure 2).

The quality of the obtained models was verified by the normality test of the residuals' distribution, statistical significance of the estimated parameters, percentage of the explained variance (>90%), and the *R* value (>0.95). The characteristics of the secretion profiles obtained for the individual TSC patients along with their DMLO estimates are gathered in Table 1.  Table 2 shows the median values of the melatonin secretion parameters as well as the DLMOs obtained for C-G and E-G.

The parameters' estimates obtained for the melatonin secretion models were subjected to statistical analysis to compare the TSC-G versus E-G group and TSC-G versus C-G group. As revealed from the Mann-Whitney-Wilcoxon tests, the secretion models' parameters for the TSC and epileptic groups do not differ statistically; however, there is a statistically significant difference between TSC-G and C-G (Table 3)—the melatonin synthesis is shifted in TSC-G patients (Figure 2). The following parameters are statistically significant: *b*_3_, DLMOon_25_, and DLMOon_50_.

Due to small sample size and apparent diversity of the melatonin secretion profiles in TSC-G, the intragroup qualitative analysis was performed (Table 1). The comparison of the received models showed that in one TSC case (Figure 1, patient 1 (red line)) with the greatest number of epileptic seizures, an obvious shift of melatonin release (4.89 h) was observed. As seen from Table 1, the TSC DLMOon and DLMOoff values obtained for patient 1 are shifted in the later hours as compared to the corresponding DLMO values for the other TSC cases.

For patient 1 (the highest amplitude), the phase shift of melatonin flux occurs later than that for C-G and for E-G. However, in two other TSC cases (Figure 1, patients 2 (green line) and 3 (blue line)), because of the seizures in the day before the examination and epilepsy associated with sleep phase, similar melatonin synthesis to that for the epileptic group could be expected (Tables 1 and 2). In the last analyzed case (Figure 1, patient 4 (magenta line)), the melatonin secretion profile was physiologically disturbed (no melatonin flux), while the clinical data did not indicate any sleep disorders or seizures.

## 4. Discussion

Recent advances in metabolomic technologies and the metabolomic studies conducted in constant routine conditions (with minimized impact of exogenous factors such as light, food, posture, and sleep) have demonstrated endogenous circadian variation in the human metabolome [11, 12]. Melatonin secretion was also found to vary per day and interpersonally, and such observation could be proven important in personalized therapies of various sleep disorders with the use of this hormone [13]. Though there are many empirical evidences of exogenous melatonin efficacy—as revealed from a big meta-study (involving a total of 5030 studies performed between 1950 and 2015)—there is still little evidence of its therapeutic usefulness from randomized, controlled experiments [14].

Children with tuberous sclerosis (TSC) often have sleep problems [15, 16]. According to Hunt [16], the authors of the first survey of 300 people with TSC (conducted in 1993), the main sleep-related problems that patients with TSC experience are settling (60%) and night waking (62%) [6]. However, the later models of disturbed sleep architecture proposed for TSC patients use different conceptual frameworks, thus, making mutual comparisons and correlations with the clinical aspects, like number of seizures, time of the onset of seizures, or the relationship of seizures to sleep, difficult [2, 17].

Our simple mathematical model has opened a tempting possibility to objectivize diurnal variations in melatonin secretion parameters and to compare them with the values obtained for the children with (E-G) and without epilepsy (C-G). Such comparisons revealed that TSC-G is similar to E-G and statistically differs from C-G. Because only four patients are in TSC-G, it is likely that we have insufficient power to detect subtle differences. The small sample size of the TSC group, the diversity of the individual melatonin profiles, and the evident influence of the outliers or extreme observations (typical in case of TSC) are the major drawbacks of our study. It may be expected that increasing the TSC group could lead to a more generalized insight into melatonin patterns, could help to obtain normal distributions of the variables, and could allow detection of more subtle changes in the melatonin secretion pattern. The small number of cases also excludes the possibility of using correlation analysis and searching other deeper relationships in the TSC group.

On the other hand, this work has demonstrated the usefulness of mathematical modeling of the secretion processes and showed that such processes can be analyzed more objectively when presented in the form of a set of parameters being easy to calculate, compare, and collect within a database. The modeling provides also possibility to estimate the dim light melatonin onset (DLMO) values. The latter is especially important in confirming sleep disorders, in differential diagnostics of several sleep disorders, and also in the studies of the influence of shift work and jetlag on human circadian rhythms [18, 19]. DLMO is also potentially valuable in studying the sleep disorders of noncircadian origin, for example, psychophysiological insomnia, bad sleep hygiene, narcolepsy, idiopathic hypersomnia, obstructive sleep apnoea, and depression-related insomnia [20].

It is interesting that all secretion patterns for the studied TSC patients differ markedly in release amplitudes—from the extremely high value being almost 3.5 times higher than the median values for E-G and C-G (patient 2) to zero for patient 4—and in phase shifts of the releases. We could not identify any factors that may have induced such high differences in the MLT amplitudes. Antiepileptic treatment, for example, valproic acid (VPA) may decrease the MLT concentration and carbamazepine (CBZ) may increase MLT metabolite excretion [16, 21]. During the study, we did not alter the existing anticonvulsant regimes; therefore, we cannot rule out the possibility that melatonin may have interacted with one or more antiepileptic drugs. It should be taken into account as well that the severity of TSC manifestation itself, varying widely between individuals, may play its role too [22, 23]. Moreover, behavioral and environmental aspects, as light-dark circle and zeitgebers influencing the circadian rhythms, like bedtime routines, temperature, social interactions, exercise, and so forth, are known to influence melatonin secretion; however, in case of the studied groups, blood collection was performed under controlled conditions.

Relationship between sleep problems and sleep-related epileptic events is often stressed in the literature [23–25]. In their early work, Hunt and Stores showed that sleep problems in TSC were strongly associated with epilepsy, the main neurological symptom of TSC [23]. Epilepsy can disrupt sleep physiology in a variety of ways [7, 9, 15, 16]. However, the sleep disturbances in TSC are likely more complex than simply being secondary to epilepsy. Neuroanatomical abnormalities, anticonvulsant medications, mental retardation, and psychiatric comorbidities, such as autism and hyperactivity, all may affect the circadian rhythms and sleep. In the only controlled polysomnographic study published so far, comparing 10 children with TSC and partial epilepsy with healthy controls, sleep architecture abnormalities (like shorter total sleep time, frequent awakenings, and decrease in REM sleep) were found in 90% of TSC children. Interestingly, sleep disturbances were seen there despite the occurrence of nocturnal seizures and the presence of EEG abnormalities [2, 22]. These authors, unfortunately, did not register the melatonin patterns. In our small TSC group, the MLT release amplitudes vary dramatically (Figure 1) and these variations seem to be independent on the total number of seizures (Table 1). However, the MLT release shift appears to depend on the number of seizures: for patient 1, with the highest total number of seizures, the secretion model is strongly disturbed and the melatonin flux is very late. In two other TSC cases (Figure 1, patients 2 (green line) and 3 (blue line)), similar circadian rhythms of melatonin synthesis to that for the epileptic group were observed, which might be due to the seizures in the day before the examination and epilepsy associated with sleep phase. The statistical analysis shows the shift of the melatonin secretion for TSC patients (DLMOon_25_, DLMOon_50_, and *b*_3_) in comparison to C-G. The results of Hancock et al. [15] on the urinal 6-sulfatoxymelatonin excretion in seven TSC patients revealed, however, no evidence of abnormal excretion of melatonin in patients with tuberous sclerosis complex and sleep disorder. All but one of the patients showed a normal circadian rhythm of melatonin secretion. But, the authors were aware that a small number of analyzed cases weakened their reasoning. Our data suggest that not only disordered sleep but also the shift of melatonin secretion may be expected in TSC children with frequent seizures; unfortunately, this supposition is based on the results gathered from a small TSC group, too. However, our previous studies performed on the large epileptic group strongly confirm it [7, 26]. Examination of melatonin secretion in patients with epilepsy has shown higher nocturnal MLT concentration, increase of MLT concentration during or after seizure, shift or loss of MLT circadian rhythm, or comparable to controls [7, 26].

## 5. Conclusion

A larger study will be needed to better understand the disturbances of circadian rhythms in TSC individuals. Based on our findings, we believe that such studies may provide more insight into the relationship between the TSC symptoms and melatonin diurnal profile. We believe that the mathematical modelling will be helpful in such task. Moreover, the presented mathematical modeling seems to be useful in estimation of the DLMO parameters.

## Figures and Tables

**Table 1:** Patients' presentation and estimated parameters of the model of melatonin secretion.

	Patient 1 5 yrs 7 mo (f)	Patient 2 5 yrs (f)	Patient 3 6 yrs (f)	Patient 4 2 yrs 10 mo (f)
Family history	Nonrelevant	Nonrelevant	Nonrelevant	Nonrelevant
Gestation, delivery period	Nonrelevant	Nonrelevant	Nonrelevant	Nonrelevant
Epilepsy	Since 6 mo	Since 3 mo	Since 5 mo	Since 6 mo
Cognitive level	Severe impairment	Severe impairment	Severe impairment	Severe impairment
EEG	Paroxysmal changes R > L	Paroxysmal changes R > L	Paroxysmal changes R > L	Paroxysmal changes with right fronto-centro-temporal predominance
Average number of epileptic seizures a day	4	1	2	1
Number of epileptic seizures a day before melatonin secretion assessment	—	1	1	—
Number of epileptic seizures a day during melatonin secretion assessment	—	—	—	—
Antiepileptic drugs (during melatonin measurement)	Valproic acid, vigabatrin	Valproic acid, vigabatrin	Valproic acid, vigabatrin, levetiracetam	Valproic acid, vigabatrin
Minimum melatonin concentration (pg/mL)	15.13	7.38	4.66	The melatonin flux did not take place
Melatonin release amplitude (pg/mL)	250.04	394.65	93.45	
Phase shift of melatonin release (h)^∗^	4.89	2.66	2.71	
Estimated sleep duration (h)^∗^	5.8	4.5	8.85	
Maximum melatonin concentration (pg/mL)	265.17	402.03	98.11	
DLMOon_50_ (h)^∗^	1.99	0.41	22.29	
DLMOoff_50_ (h)^∗^	7.8	4.91	7.14	
DLMOon_25_ (h)^∗^	1.20	23.79	21.08	
DLMOoff_25_ (h)^∗^	8.59	5.53	8.56	

^∗^Time in a decimal system.

**Table 2:** Average melatonin secretion parameters in C-G and E-G.

	C-G	E-G
Minimum melatonin concentration (pg/mL)	8.37	7.76
Melatonin release amplitude (pg/mL)	117.98	114.03
Phase shift of melatonin release (h)^∗^	2.0	2.57
Estimated sleep duration (h)^∗^	6.87	6.51
Maximum melatonin concentration (pg/mL)	126.35	121.79
DLMOon_50_ (h)^∗^	22.57	23.32
DLMOoff_50_ (h)^∗^	5.44	5.83
DLMOon_25_ (h)^∗^	21.63	22.43
DLMOoff_25_ (h)^∗^	6.38	6.72

^∗^Time in a decimal system.

**Table 3:** *p* values for the Mann-Whitney-Wilcoxon tests obtained for the TSC-G and C-G comparison (the statistically important values are in bold).

Parameters	*p*
*b*_1_—minimum melatonin concentration	0.730528
*b*_2_—melatonin release amplitude	0.510837
*b*_3_—phase shift of melatonin release	**0.041829**
*b*_4_—sleep duration (FWHM)	0.551902
*b*_max_ given by a sum of *b*_1_ and *b*_2_	0.510837
DLMOon_25_	**0.035919**
DLMOoff_25_	0.331725
DLMOon_50_	**0.022271**
DLMOoff_50_	0.246655
